# Chronic Intestinal Pseudo-Obstruction in a Young Male With Alcohol Abuse Disorder

**DOI:** 10.7759/cureus.13824

**Published:** 2021-03-11

**Authors:** Asim Haider, Shehriyar Mehershanhi, Ayesha Siddiqa, Harish Patel

**Affiliations:** 1 Internal Medicine, BronxCare Health System, Bronx, USA; 2 Gastroenterology and Hepatology, BronxCare Health System, Bronx, USA; 3 Gastroenterology and Hepatology, BronxCare Health System, New York, USA

**Keywords:** chronic intestinal pseudo-obstruction, cipo, intestinal dilatation

## Abstract

Chronic intestinal pseudo-obstruction (CIPO) is a rare syndrome associated with significant mortality and morbidity. It mimics the signs and symptoms of intestinal obstruction in the absence of an anatomic lesion causing obstruction. Here we present a case of a young male with severe alcohol abuse disorder who initially presented with signs and symptoms of alcohol withdrawal but was found to have abdominal distension. Imaging studies revealed severe small and large bowel dilatation without any organic lesion. He continued to have marked intestinal dilatation for the subsequent few months. Alcohol cessation eventually led to a marked reduction in his symptoms and a decrease in intestinal dilatation. The occurrence of CIPO because of alcohol abuse is rare, and we explore the possible association between the two entities.

## Introduction

Pseudo-obstruction is a syndrome characterized by signs and symptoms of mechanical obstruction of the large or small bowel in the absence of an anatomic lesion. It may be acute or chronic and is characterized by dilation of the bowel on imaging. The term chronic pseudo-obstruction should be differentiated from a related disorder called intestinal dysmotility, characterized by the evidence of chronic small intestinal motility disorder in the absence of bowel dilatation. Chronic intestinal pseudo-obstruction (CIPO) is rare. Iida et al. reported an incidence of 0.21-0.24 per 100,000 and a prevalence of 0.80-1.00 per 100,000 in a study conducted in Japan [1]. The mean age at diagnosis was 63 years for males and 59 for females in the same study. Here we present a case of a young male who developed CIPO due to severe alcohol abuse disorder.

## Case presentation

**Figure 1:**
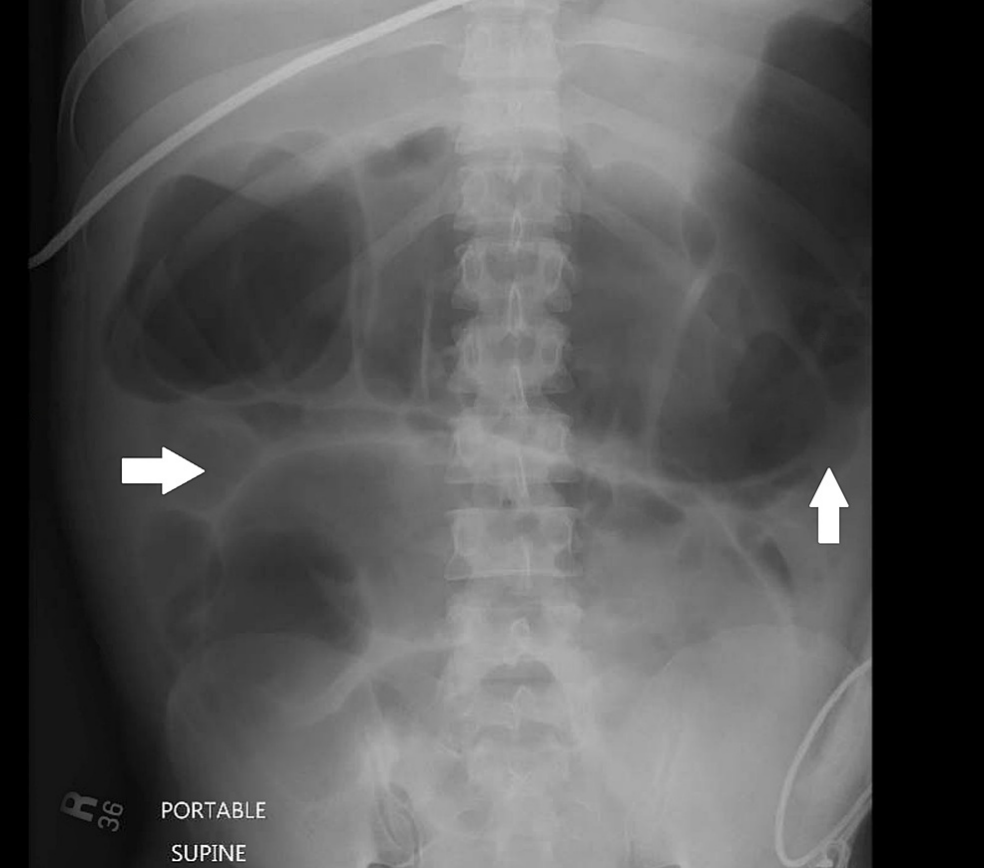
X-ray abdomen showing marked gaseous distension of the stomach and intestinal loops.

A 35-year-old male with a medical history significant for severe alcohol abuse disorder presented to the ED with altered mental status, nausea, and vomiting for one day. He had been drinking almost more than a gallon of alcohol per day for months, but his last drink was two days ago. He also reported multiple episodes of yellowish watery diarrhea for the previous few weeks. He had no known history of cardiovascular, pulmonary, metabolic, neurological, autoimmune, inflammatory disease, or any previous surgery. He was not taking any medications. On physical examination, the patient was alert and oriented to place and person but not to date. His abdomen was soft, diffusely distended, but nontender. He was tachycardiac to 120 s, but the rest of the vital signs were within normal limits. The laboratory findings showed hemoglobin of 14 g/dL, white blood cells 6,210/mm3, and platelets 300,000/mm3. He was managed for the alcohol withdrawal initially, but over the course of his stay in the hospital, he continued to complain of yellowish watery diarrhea with four to five episodes per day. Stool work up including stool ova and parasite, culture, fecal leukocyte esterase, fecal fat, fecal calprotectin, and clostridium difficile toxin were negative. The celiac panel, thyroid panel, antinuclear antibodies (ANA), erythrocyte sedimentation rate (ESR), C-reactive protein (CRP), serum/urine immunofixation, and hepatitis serology were also negative. X-ray of the abdomen marked dilatation of small and large bowel loops (Figure 1).

**Figure 2:**
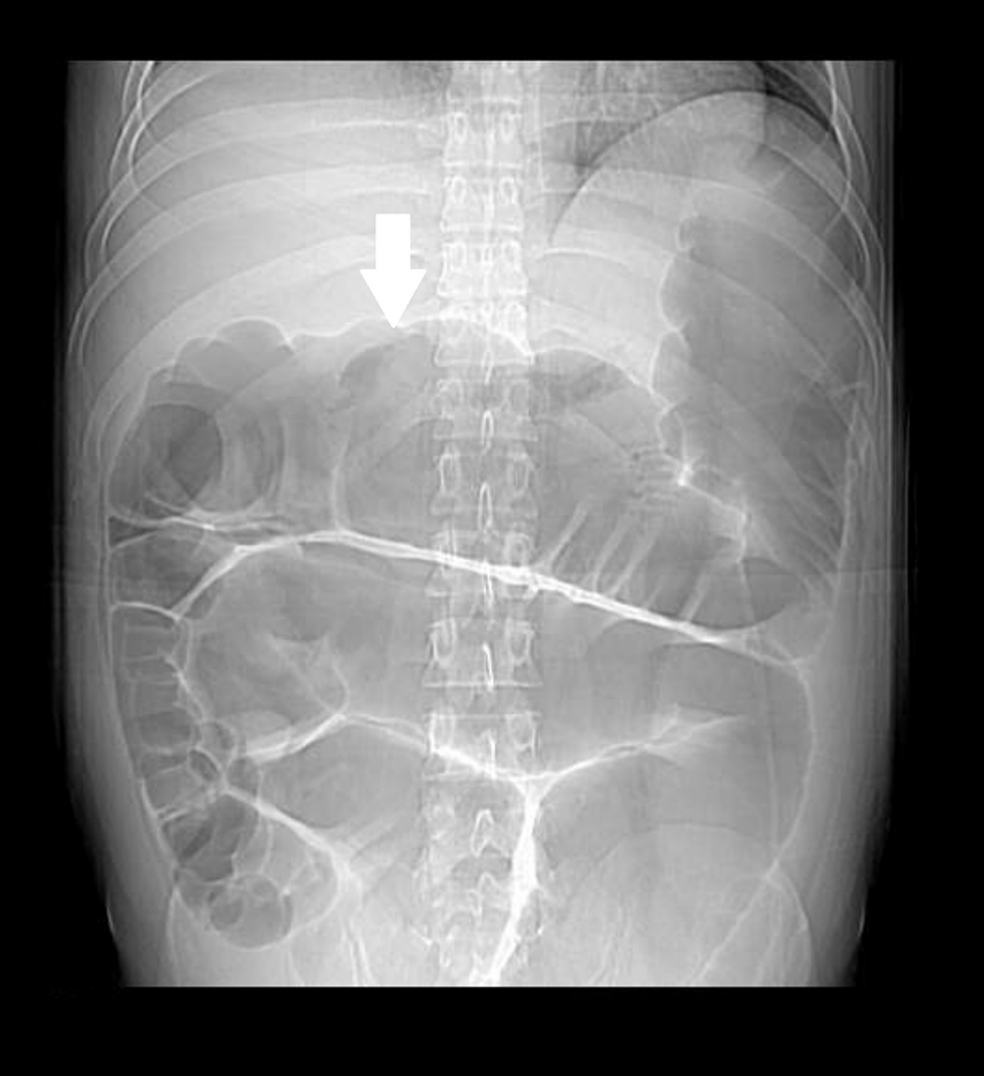
CT scan of the abdomen showing multiple distended small and large bowel loops.

CT scan of the abdomen showed multiple distended small and large bowel loops with gas. No transition point was seen to suggest any mechanical obstruction (Figure 2).

**Figure 3:**
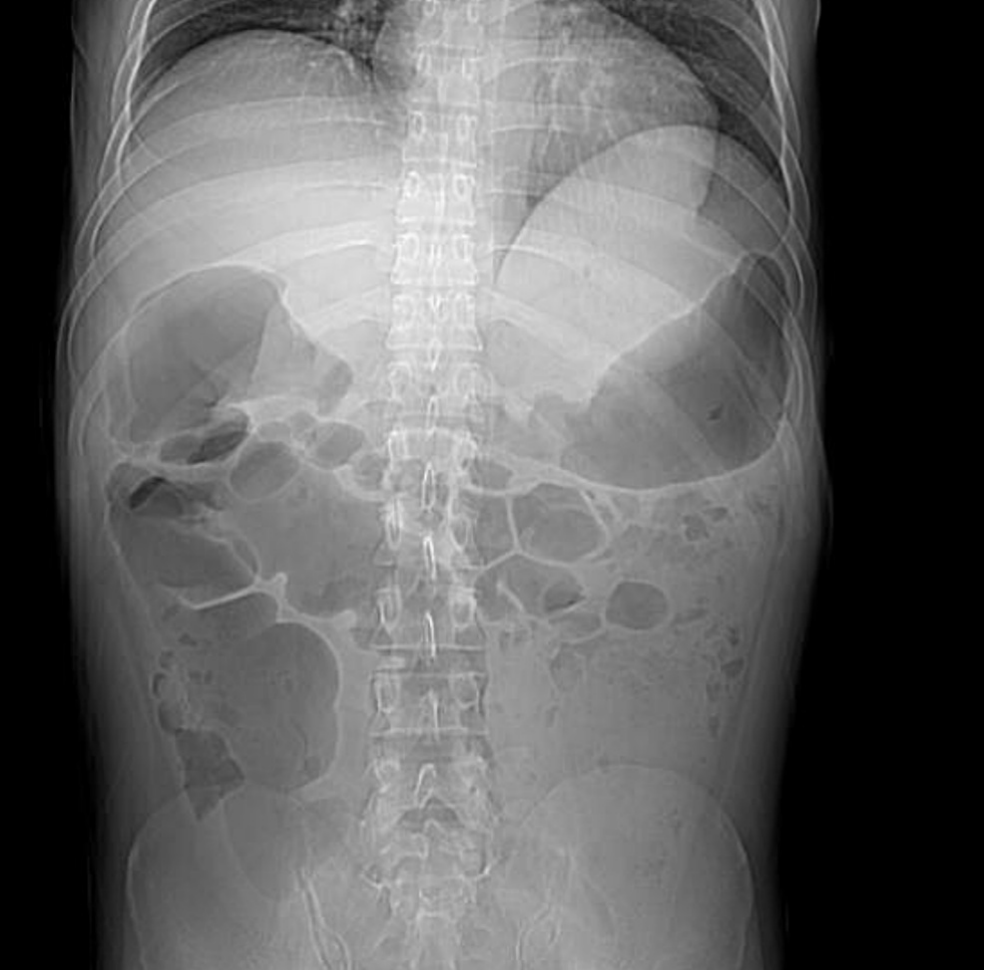
Follow up CT scan of the abdomen and pelvis showing significantly decompressed colon.

He underwent colonoscopy, which showed a grossly normal colon. Random biopsies were taken from the colon, which was unremarkable. A push enteroscopy showed a single nonbleeding ulcer in the lower third of the esophagus (biopsy result: negative). The examined portions of the duodenum and jejunum were grossly normal. Random biopsies taken from the small intestine were also negative. In view of the distended intestine with essentially extensive negative workup, the diagnosis of CIPO was made. The diarrhea was attributed to small intestinal bacterial overgrowth. A trail of rifaximin was given, which led to an improvement in diarrhea. The patient was counseled about the alcohol cessation and was discharged to an alcohol rehabilitation facility. Four months later, the patient was seen in the outpatient clinic with almost complete diarrhea resolution, abdominal distension, and bloating. A follow-up CT scan of the abdomen and pelvis showed a significantly decompressed colon (Figure 3). 

## Discussion

The pathophysiological mechanism underlying the CIPO may be an underlying neuropathic disorder (involving the enteric nervous system or extrinsic nervous system), a myopathic disorder (involving the smooth muscle), or an abnormality in the interstitial cell of Cajal (ICC) [2]. Neuropathic, myopathic, or ICC abnormalities may be idiopathic or secondary to another disease. There are several etiologies described in the literature as the possible culprits causing CIPO via the mechanisms mentioned above. These include neurological disordered (e.g., Parkinson disease and Shy-Drager syndrome), metabolic disorders (e.g., diabetes mellitus), paraneoplastic (e.g., small cell lung cancers, carcinoid tumors [3], malignant thymoma [4], and prostate cancer [5]), immune medicated (scleroderma, dermatomyositis, and systemic lupus erythematosus [6]), infectious (e.g., Chagas disease, viruses [7]), and in association with radiotherapy and chemotherapy for cancers. 

Chronic alcohol abuse has long been known to cause several neurological disorders, including confusion, cerebellar ataxia, central and peripheral neuropathy. Alcohol-related neuropathy can involve small as well as large nerve fibers [8]. The severity of neuropathy is dose-related [9]. The pathogenesis of neuropathy related to alcohol is still not clear. Earlier, nutritional deficiencies, especially thiamine deficiency was considered the reason for neuropathy in alcoholics. Recent studies suggest a rather direct neurotoxic effect of alcohol and its metabolites. A direct neurotoxic effect of alcohol was observed in animal models while monitoring normal thiamine levels [10]. At the same time, alcohol also causes both skeletal and smooth muscle myopathy. We believe that these neuropathic and myopathic properties of alcohol lead to the development of severe CIPO in our patients. 

Abdominal pain, bloating, and distension are the most common clinical features of CIPO. The symptoms may be acute, recurrent, or chronic. Acute episodes are characterized by rapid onset of severe crampy pain, abdominal distention associated with nausea and vomiting. In between the acute episodes, patients may be asymptomatic or continue to experience symptoms. The symptoms may be due to delayed transit in the upper gastrointestinal tract (e.g., anorexia, early satiety, nausea, and vomiting) or distal tract (constipation). These patients may have diarrhea due to small bowel bacterial overgrowth (as seen in our patient). 

The diagnosis of CIPO is based on the presence of long-standing mechanical obstruction symptoms in the absence of an anatomic cause on radiological examination and endoscopy and evidence of impaired motility. Patients should undergo an initial evaluation with radiographic testing to exclude organic causes of obstruction (e.g., plain radiographs and contrast imaging). Upper gastrointestinal endoscopy and colonoscopy should be performed to rule out an intraluminal or extraluminal cause of obstruction. In patients in whom CIPO is suspected, and there is no evidence of an intraluminal or extraluminal cause of obstruction on imaging, and by endoscopy, the presence of a motility disorder should be confirmed with scintigraphy which is the method of choice in the evaluation of gastric, small bowel, and colon transit.

A multidisciplinary team is recommended for the management of CIPO, including the surgeon, gastroenterologist, and nutritionist [11]. These patients require supplemental nutritional support. Prokinetic agents, such as erythromycin and cisapride, may be used for acute and CIPO therapy, respectively [12]. However, these treatments are off-label, and risks and benefits should be discussed with the patient. The combined use of oral cisapride and erythromycin can lead to significant arrhythmia (torsades de pointes) and should be avoided. Prucalopride (a 5HT4 receptor agonist) accelerates transit through the gastrointestinal tract and has shown benefit in CIPO [13]. Acute exacerbations can also be treated with metoclopramide and anticholinesterases (e.g., neostigmine, pyridostigmine) [14]. Patients with CIPO who have steatorrhea, vitamin B12 malabsorption, or folate excess may have bacterial overgrowth and should be treated empirically with antibiotics.

## Conclusions

Chronic intestinal pseudo-obstruction is a syndrome characterized by signs and symptoms mimicking intestinal obstruction in the absence of an organic lesion. The underlying mechanism could be a neuropathic or myopathic disorder (involving the smooth muscle) or an abnormality in the ICC. We discussed a case of a young male with severe alcohol abuse disorder who presented with clinical and radiological features of CIPO. Alcohol cessation led to a marked improvement in his symptoms. The association between alcohol abuse and CIPO needs to be explored by further studies. 
